# Assessing Normandy Soil Microbial Diversity for Antibacterial Activities Using Traditional Culture and iChip Methods

**DOI:** 10.3390/microorganisms12122422

**Published:** 2024-11-25

**Authors:** Fabien Perrier, Juliette Morice, Sabrina Gueulle, Antoine Géry, Eliette Riboulet-Bisson, David Garon, Cécile Muller, Florie Desriac

**Affiliations:** 1 Université de Caen Normandie, CBSA UR 4312, UFR des Sciences, Campus 1, F-14000 Caen, France; juliette.morice@unicaen.fr (J.M.); sabrina.gueulle@unicaen.fr (S.G.); eliette.bisson@unicaen.fr (E.R.-B.); cecile.muller@unicaen.fr (C.M.); 2 Université de Caen Normandie, ToxEMAC-ABTE UR 4651, UFR des Sciences, Campus 1, F-14000 Caen, France; antoine.gery@unicaen.fr (A.G.); david.garon@unicaen.fr (D.G.)

**Keywords:** antimicrobial resistance, iChip, in situ cultivation, uncultured bacteria, fungi, antibacterial activity, microbial natural product

## Abstract

Uncultured microorganisms represent a promising and untapped source of antibacterial compounds, crucial in the fight against the significant threat of antimicrobial resistance (AMR). In this study, both traditional and isolation chip (iChip) cultivation techniques were employed to enhance the recovery of known and unknown microorganisms from soils located in Normandy, France. The isolates obtained were identified using 16S rDNA or ITS regions analysis and MALDI-TOF mass spectrometry and were screened for antibacterial activity. A total of 386 isolates, belonging to 6 microbial phyla and distributed across 65 genera, were recovered using both methods. In total, 11 isolates are potentially new bacterial species, and 34 were associated with 22 species described recently. The iChip method yielded a higher diversity of microorganisms (47 genera) than the traditional method (38 genera) and was particularly effective in enriching *Actinomycetota*. Antibacterial screening against target bacteria showed that 85 isolates (22%) exhibited antibacterial activity. The *Streptomyces*, *Pseudomonas*, and *Bacillaceae* taxa accounted for most antibacterial-producing bacteria with some presenting promising undescribed characteristics. Other active isolates were affiliated with less-known antibacterial producers such as *Arthrobacter*, *Chryseobacterium*, *Delftia*, *Ensifer*, *Flavobacterium*, *Rahnella*, and *Stenotrophomonas*, among others. These results highlight the potential of our microbial collection as a source of new antibacterial natural products.

## 1. Introduction

Antimicrobial resistance (AMR) was responsible for an estimated 1.27 million global deaths in 2019 and represents a major threat to human, animal, and environmental health [1]. If insufficient action is undertaken, alarming projections suggest that the AMR burden could result in 39.1 million cumulative deaths attributable to AMR and 169 million deaths associated with AMR worldwide by 2050 [2], with a cumulative cost of USD 100 trillion to society [3]. To tackle this issue, the World Health Organization (WHO) has published a list of drug-resistant bacterial pathogens to guide research priorities and the development of new antibacterial agents [4]. The clinical pipeline for antibacterial drugs increased slightly between 2021 and 2023 with 46 and 57 traditional antibiotics being developed, respectively [5,6]. However, this progress remains largely insufficient to tackle the challenge of the increasing emergence and spread of AMR. Consequently, the continued discovery of new molecules with antibacterial activity is urgently needed to fight the AMR crisis.

Microbial natural products are the most prolific source of antibacterial agents, with 67% of all FDA-approved antibacterial being microbial secondary metabolites or their derivatives [7]. Most antibiotic classes were found between the 1940s and the 1970s from empirical screening of soil-dwelling microorganisms, particularly the bacterial phylum *Actinomycetota*, from which around two-thirds of all known antibiotics are derived [8,9,10,11]. Then, despite intensive worldwide efforts using alternative approaches such as combinatorial chemistry and high-throughput screening or artificial intelligence, no other concept has thus far surpassed the use of microorganisms’ metabolites as candidates for anti-infective drugs [11,12]. The microbial reservoir seemed depleted after discovering the last natural class of antibiotics, the lipopeptides, with daptomycin in 1987 [10,13]. This dry-up has been mainly attributed to the screening of historical producers such as *Streptomyces* resulting in the isolation of already-known chemical entities [14]. Since then, no new antibiotic classes have been developed from natural origins [14].

Yet, microorganisms remain a promising source for antibiotic drug discovery. Indeed, recent advances in genome-sequencing technologies and computational algorithms have revealed the biosynthetic potential of microbial genomes, which harbor a wide diversity of biosynthetic gene clusters (BGCs) with no final product identified [15,16,17]. Additionally, metagenomic and phylogenetic studies have shown that microbial diversity has been vastly underestimated in past decades. It is well established that less than 1% of microorganisms on Earth are cultivable in laboratory settings, and several microbial lineages have few or no cultivated representatives [18,19,20,21]. Exploration of the uncultured microbial majority, also known as ‘microbial dark matter,’ therefore holds great promise for the discovery of new biotechnological compounds, especially new antibiotics.

Uncultured microorganisms are non-growing microbes in laboratory conditions for which the growth requirements are still to be determined. These needs may include precise nutrition and growth factors, physical factors such as temperature, pH, and oxygen level, or symbiotic/syntrophic relationships with other organisms. These microorganisms may also be slow growers, low-abundant, or dormant, explaining their uncultivability [21,22,23]. Many cultivation efforts have been made to favor the recovery of these microorganisms: changes in media composition (nutrients, concentrations, addition of specific factors, …), application of physical or chemical treatments, changes in the incubation parameters (temperature, pH, salinity, oxygen level, incubation time, …), culturomics, and co-culture among others, with relative success [21,22,23,24,25]. However, since no general criteria can be applied to access this diversity, cultivating the hitherto uncultured microorganisms remains challenging and laborious.

At the beginning of the 2010s, a promising technique known as the isolation chip (iChip) was created to facilitate the domestication of novel microorganisms by simulating their natural environment [26]. The iChip is known for the domestication of the previously uncultured bacterium *Eleftheria terrae*, which produces teixobactin and clovibactin, two innovative antibiotics with new targets and active against certain Gram-positive bacteria including methicillin-resistant *Staphylococcus aureus* (MRSA) and vancomycin-resistant enterococci (VRE) strains [27,28]. This technology involves microbial cells being trapped in multiple wells filled with agar, placed between semi-permeable membranes, and incubated in the original environment. The required nutrients and growth factors present in the surrounding environment can diffuse into the device to support the growth of microorganisms. This enables their ability to grow ex vivo (domestication) [26,29].

Genomic and metagenomic data, as well as assessing the hitherto uncultured microorganisms, reinvigorate the quest for new antibacterial compounds produced by microorganisms. To access a higher diversity of microorganisms, we employed recognized methods: direct plating cultivation on a variety of media and the iChip technology. Both methods were performed on diverse soils from Normandy, France. The obtained isolates were identified using 16S rDNA or ITS (Internal Transcribed Spacer) regions’ analysis and matrix-assisted laser desorption/ionization time-of-flight (MALDI-TOF) mass spectrometry. Finally, isolates were screened for antibacterial activity against target microorganisms according to the WHO critical list [4].

## 2. Materials and Methods

### 2.1. Soil Sampling

Nine soil samples (S1–S9) were collected between November 2022 and June 2023 at different locations in Normandy, France (Table S1). The samples were taken from approximately 20 cm below the soil surface (except for S7 at 50–100 cm) using a garden shovel pre-treated with ethanol and washed with sterile pure water. Around 2 kg of soil was placed in UV-pre-treated 2 L containers and stored at room temperature until use for a maximum of 3 days.

### 2.2. Cultivation Methods

#### 2.2.1. Isolation Chips

The inoculation and incubation of iChips were adapted from the protocol of Berdy et al. [29]. Briefly, soil slurry was generated from each soil sample. In total, 1g of soil, sifted through a 0.3 cm wire mesh, was mixed with 10 mL of sterilized 0.9% (*w*/*v*) NaCl (Honeywell, Saint-Germain-en-Laye, France) solution (saline solution) and crushed using a sterile mortar and pestle. To optimize the dissociation of microbial cells from the environmental matrix, the slurry was vortexed for 10 min followed by 10 min of decantation. The microbial cell density of the supernatant was estimated by enumeration using a Thoma counting chamber under optical microscopy (X400–X1000, Primostar, Zeiss, Oberkochen, Germany). The supernatant was diluted in saline solution and mixed with molten agar (Solabia, Pantin, France) at a concentration of 10 g/L, 40 °C, to achieve a cell density of 1 to 10 cells per 200 µL. Two hundred microliters of cells-agar mixture was distributed in a 96-well iChip frame (200 µL pipet tips rack, ClearLine^®^, Bernolsheim, France) that had been pre-glued (silicone glue SA 500, Zolux, Saintes, France) with a polycarbonate track-etched (PCTE) 0.03 µm semi-permeable membrane (GVS Filter Technology, Bologna, Italy) at the bottom. After agar solidification, a second membrane was applied to the top to seal the device.

**Figure 1:**
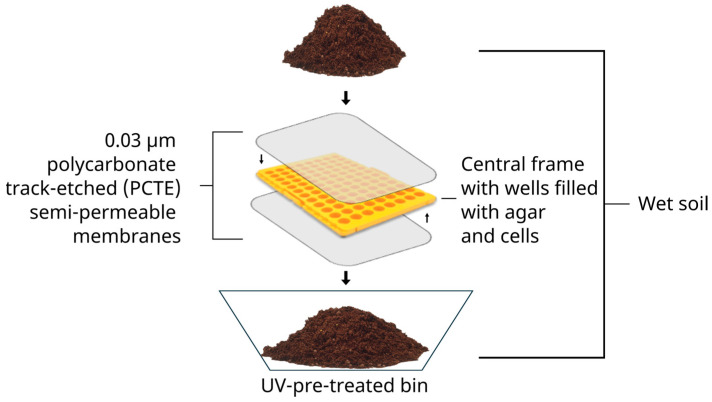
In situ cultivation of microorganisms using isolation chip (iChip) technology. The iChip device is composed of a central frame constituted of 96 wells filled with 1–10 cells in agar, and two 0.03 µm PCTE semi-permeable membranes stuck on both sides. The iChip device is placed in a wet soil bin to simulate the natural environment of microorganisms. Nutrients and growth factors diffuse across the pores of the membranes to support cell growth.

The iChips were buried in the initial soil placed in a UV-pre-treated bin (18 × 18 × 8 cm) to simulate the natural environment (Figure 1). In situ incubation was conducted in the laboratory at 23 °C in the dark for 1, 2, or 3 weeks. The humidity was maintained by periodically spraying sterile pure water on the soil surface.

After incubation, iChips were recovered and washed with sterile water to remove any soil debris. Once dried, the devices were opened, and each agar plug was transferred into 300 µL of saline solution using sterile tips. The agar plugs were disrupted with 1 mL syringes (Terumo, Tokyo, Japan), and 100 µL of the resulting mixture was plated on LB agar (Lysogeny Broth: 10 g/L tryptone (Solabia), 5 g/L yeast extract (Solabia), 5 g/L NaCl (Honeywell), and 15 g/L agar or diluted 1/5 LB agar media). The plates were incubated at 23 °C in the dark for 7 days. Colonies were selected based on their macroscopic appearance and serially streaked onto fresh agar plates until pure cultures were obtained. Additionally, the isolates were cultivated in liquid cultures (120 rpm, 23 °C) for 72 h and cryopreserved in LB or diluted 1/5 LB without agar supplemented with 15% (*v*/*v*) glycerol, at −20 °C and −80 °C. Prior to identification, isolates were designated as SX-CY-Z (SX: sample number, CY: number of in situ incubation weeks, and Z: isolate number).

#### 2.2.2. Dilution and Direct Plating (DDP)

From each soil-slurry supernatant generated for the inoculation of iChips (as described above), serial dilutions down to 10^−6^ were prepared in saline solution and 100 µL was spread onto the following agar media: LB, diluted 1/5 LB, TSB (Tryptone Soy Broth; Oxoid, Basingstoke, UK), and diluted 1/5 TSB. Plates were incubated at 23 °C in the dark for 7 days. Pure cultures were obtained by streaking selected colonies onto fresh agar plates. Isolates were then cultivated for 72 h in liquid cultures (120 rpm, 23 °C) in their recovery medium without agar and supplemented with 15% (*v*/*v*) glycerol, before storage at −20 °C and −80 °C for long-term preservation. Prior to identification, isolates were designated SX-Z (SX: sample number, and Z: isolate number).

### 2.3. Microbial Identification and Phylogenetic Analysis

#### 2.3.1. MALDI-TOF Mass Spectrometry Identification

Isolates were cultivated on their respective recovery agar medium for 24 to 72 h at 23 °C to produce small colonies. After incubation, microbial material from a single isolated colony was collected and deposited on an MTP 96 MALDI-TOF target plate (Bruker Daltonics, Leipzig, Germany). The sample was then overlaid with 1 µL of α-cyano-4 hydroxycinnamic acid matrix solution (Bruker Daltonics) and the matrix-sample was crystallized by air-drying at room temperature for 5 min. The identification process was completed using a MALDI Biotyper^®^ Sirius System (Bruker Daltonics).

Identification results at the genus or species level were accepted according to Bruker’s instructions. A score in the range of 2.00–3.00 indicates a high confidence in identification at the species level. Identification at the genus level is accepted for a score of 1.7–1.99 (low confidence identification at the species level). No organism identification is possible for scores below 1.7.

#### 2.3.2. Molecular Identification

Most isolates showing antibacterial activity or with no identification via MALDI-TOF analysis were identified using 16S rDNA or ITS region sequencing.

From fresh agar culture, microbial material was transferred into 250 µL of saline solution to prepare a cell or mycelium suspension, and 0.3 mm microbeads were added. The suspension was lysed using a Mixer Mill MM200 (Retsch GmbH, Hann, Germany) at a frequency of 30 Hz for 7 min 30 s for non-mycelial strains, and 15 min for mycelial strains. Lysate was used as a template for PCR amplification. The bacterial 16S rDNA was amplified using the universal primers 27F (5′-AGAGTTTGATCCTGGCTCAG-3′) and 1492R (5′-TACGGYTACCTTGTTACGACTT-3′) [30]. For fungi, the primers ITS1F (5′-CTTGGTCATTTAGAGGAAGTAA-3′) and ITS4 (5′-TCCTCCGCTTATTGATATGC-3′) were used to amplify ITS regions [31,32]. Since no PCR products were obtained for S1-C2-9 and S7-C3-10 fungal isolates, identification was performed by hyphae and conidia observation at the macroscopic and microscopic scales under a microscope after a 7-day culture on malt extract (Oxoid, Basingstoke, UK) agar medium. GoTaq DNA Polymerase (Promega, Madison, WI, USA) was used for the PCR reactions following the manufacturer’s recommendations. Amplicons were purified with the QIAquick^®^ PCR purification kit (Qiagen, Hilden, Germany) following the kit instructions and quantified using a Nanodrop™ One system (Thermo Fisher Scientific, Waltham, MA, USA). The purified amplicons were then sequenced by the Sanger method at Eurofins Genomics (Cologne, Germany) using the same primers as for PCR amplification. Generated sequences were quality-checked, paired, and the sequence ends were trimmed using SnapGene software v7.1.1. Sequences were submitted to NCBI GenBank with the accession numbers: PQ395031 to PQ395179 and PQ433420 to PQ433424 for 16S rDNA sequences, PQ400030 to PQ400037 for ITS regions sequences (Table S2).

Assignation was made using the Basic Local Alignment Search Tool (BLAST) of the National Center for Biotechnology Information (NCBI) (https://blast.ncbi.nlm.nih.gov/Blast.cgi, accessed on 22 June 2024) to determine the phylogenetic neighbors in the GenBank database. For each isolate, the sequence of the closest type strain was retrieved from the database. Multiple sequence alignment was performed with the ClustalW algorithm and phylogenetic trees were built by the neighbor-joining and maximum-parsimony methods using Mega software v11.0.13 [33,34,35,36]. The topologies of the trees were evaluated using the bootstrap method based on 1000 replicates [37]. Kimura’s two-parameter model was used to analyze the evolutionary distances among the strains [38]. *Azotobacter nigricans* IAM 15005^T^, *Kitasatospora cheerisanensis* YC75^T^, and *Paenibacillus polymyxa* DSM 36^T^ were used as outgroups for the *Pseudomonas*, *Streptomyces*, and *Bacillaceae* trees, respectively.

### 2.4. Indicator Strains and Culture Conditions

Antibacterial screening of the microbial collection was conducted against the following indicator strains: two clinical isolates, *Klebsiella pneumoniae* 2020901 (KP) and *Enterococcus faecalis* JH2-2 (EF); and four collection strains, *Micrococcus luteus* ATCC 10240 (ML), *Escherichia coli* CIP 54127 (EC), *Staphylococcus aureus* UCNU 7734 (SA), and *Pseudomonas aeruginosa* CIP A22 (PA). All strains are cryopreserved in glycerol and stored in a freezer at −80 °C. ML was chosen for its known sensitivity to a wide variety of antibiotics. The other indicator strains were selected due to their species being reported on the WHO bacterial priority pathogens list [4]. SA and EF were grown in TSB, while the other strains were grown in LB. All target bacteria were grown at 37 °C, 120 rpm, except for EF, which was grown under static conditions.

### 2.5. Antibacterial Screening

#### 2.5.1. Deferred Antagonism Assays

A freshly isolated environmental colony was transferred onto a 15 mL layer of agar according to its recovery media (Table S4) using a sterile toothpick. Plates were incubated at 23 °C for 3 or 7 days until satisfactory spot growth was achieved. In parallel, indicator strains were grown overnight (~16 h) in their respective media (described in Section 2.4). Fresh media were then inoculated with these cultures at 1% (*v*/*v*) and incubated at 37 °C. When the optical density OD_600 nm_ reached 0.5, cultures were diluted in molten agar media (40 °C, 15 g/L) to obtain 1 × 10^6^ CFU/mL, and 15 mL was gently poured over the spots of the screened isolates. After agar solidification, plates were incubated (37 °C, 24 h). The diameters of the inhibition zones around the spots were then measured and categorized as follows: (−): no inhibition, (+): inhibition diameter < 0.4 cm, (++): 0.4 cm ≤ inhibition diameter < 1 cm, (+++): inhibition diameter ≥ 1 cm.

#### 2.5.2. Liquid Fermentation and Agar-Well Diffusion Assays

Isolates were grown in their recovery broth medium (72 h, 23 °C, 120 rpm; Table S5). Then, 10 or 100 mL of fresh medium was inoculated at 1% and cultures were incubated at 23 °C for 3 or 7 days at 120 rpm or 7 days under static conditions. Cultures were then centrifuged (10 min, 10,000 rpm). In parallel, indicator strains were prepared as described in Section 2.5.1. Fifty microliters of the culture supernatants of the screened isolates was deposited into 0.5 cm-diameter wells in agar plates. Plates were incubated and the diameters of inhibition zones around the wells were measured and categorized as described in Section 2.5.1. If no antibacterial activity was observed, supernatants were freeze-dried using a Christ Alpha 2-4 freeze-drier (Martin Christ GmbH, Osterode, Germany). The resulting powders were suspended in a minimal volume of sterile pure water, allowing a 10- to 20-fold increase in microbial metabolite concentration, and were re-tested against indicator strains.

## 3. Results

### 3.1. Microbial Diversity from Normandy Soils

Nine environmental soil samples (S1–S9) were collected in Normandy, France (Table S1). To maximize microbial diversity, samples were taken from locations with varying levels of human influence, with samples S3 and S7 from environments with high anthropic impact and samples S1, S2, S4, S5, S6, S8, and S9 from areas with minimal human impact. In addition, microbial colonies were picked according to their morphological aspects on plates for each cultivation method, without considering the colony appearance of microorganisms cultivated by the other method. A total of 386 isolates were retrieved, 182 were obtained using DDP and 204 isolates using the iChip method. Among the 386 isolates, 338 were identified through MALDI-TOF mass spectrometry and/or sequencing of the 16S rDNA or ITS regions (158 and 180 identified isolates for DDP and the iChip, respectively, Table S2). Forty-eight microbial isolates were not identified either due to the low viability of the isolates or negative PCR amplification, despite several attempts. These isolates originated from the DDP or iChip methods, with no distinction between them. The 16S rDNA or ITS region sequences of 162 isolates were deposited in NCBI’s GenBank database (Table S2). At the phylum level, no specificity was observed based on the soil’s origin. Indeed, phyla were recovered regardless of whether the samples were collected from an urban area (S3 and S7), meadows (S1, S4, S5, S6, and S9), or near a lake (S2 and S8).

**Figure 2:**
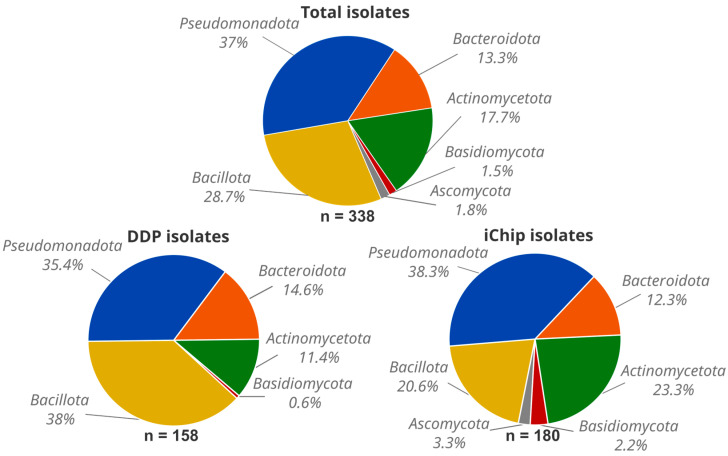
Microbial diversity of cultivated isolates at the phylum level for all recovered microorganisms (total) or by cultivation method: dilution and direct plating (DDP) and iChip. n: number of identified isolates.

From our environmental samples, *Pseudomonadota* was the most abundant phylum (125 isolates, 37%), followed by *Bacillota* (97 isolates, 28.7%), *Actinomycetota* (60 isolates, 17.7%), *Bacteroidota* (45 isolates, 13.3%), *Ascomycota* (6 isolates, 1.8%), and *Basidiomycota* (5 isolates, 1.5%) (Figure 2). In our study, the microbial phyla proportions appeared to be independent of the origin of the soil samples. *Pseudomonadota* and *Bacteroidota* were found in similar relative abundances with both the DDP and iChip methods (*Pseudomonadota:* 56 isolates, 35.4%, and 69 isolates, 38.3%; and *Bacteroidota*: 23 isolates, 14.6%, and 22 isolates, 12.3%, respectively). In contrast, *Bacillota* isolates were more abundant with DDP (60 isolates, 38%) compared to the iChip (37 isolates, 20.6%), whereas *Actinomycetota* were enriched using the iChip (42 isolates, 23.3%) compared to DDP (18 isolates, 11.4%). Additionally, all fungal isolates belonging to *Ascomycota* (6 isolates, 1.8%) and *Basidiomycota* (4 isolates, 1.2%) were retrieved using the iChip, except for one *Basidiomycota* isolate (S9-31) (Figure 2 and Table S2).

**Figure 3:**
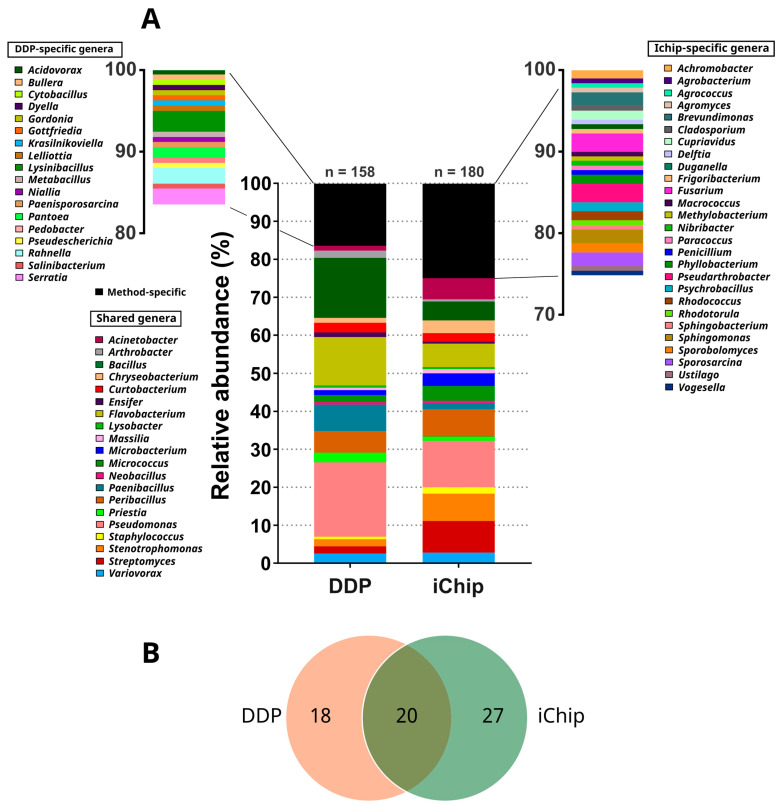
(**A**) Relative abundances of the 65 genera for DDP and the iChip. n: number of identified isolates. (**B**) Venn diagram of the number of shared and exclusive genera for the two cultivation methods.

At the genus level, isolates were classified into 65 different genera (Figure 3A). The most represented genus was *Pseudomonas* (53 isolates, 15.7%), followed by *Bacillus* (34 isolates, 10.1%), *Flavobacterium* (31 isolates, 9.2%), *Peribacillus* (22 isolates, 6.5%), *Streptomyces* (18 isolates, 5.3%), *Stenotrophomonas* (16 isolates, 4.7%), *Paenibacillus* (14 isolates, 4.1%), *Acinetobacter* (12 isolates, 3.6%), and *Micrococcus* (10 isolates, 3%). Furthermore, two or more isolates belonged to the following genera: *Variovorax* (nine isolates, 2.7%)*, Chryseobacterium*, *Curtobacterium*, *Microbacterium* (each with eight isolates, 2.4%), *Priestia* (six isolates, 1.8%), *Arthrobacter*, *Fusarium*, *Lysinibacillus*, *Pseudoarthrobacter*, *Staphylococcus* (each with four isolates, 1.2%), *Brevundimonas*, *Ensifer*, *Massilia, Rahnella*, *Serratia*, *Sphingomonas*, *Sporosarcina* (each three isolates, 0.9%), *Achromobacter*, *Cupriavidus*, *Lysobacter*, *Neobacillus*, *Pantoea*, *Phyllobacterium*, *Psychrobacillus*, *Rhodococcus*, and *Sporobolomyces* (each with two isolates, 0.6%). From the genera *Acidovorax*, *Agrobacterium*, *Agrococcus*, *Agromyces*, *Bullera*, *Cladosporium*, *Cytobacillus*, *Delftia*, *Duganella*, *Dyella*, *Frigoribacterium*, *Gordonia*, *Gottfriedia, Krasilnikoviella*, *Lelliottia*, *Macrococcus*, *Metabacillus*, *Methylobacterium*, *Niallia*, *Nibribacter*, *Paenisporosarcina*, *Paracoccus*, *Pedobacter*, *Penicillium*, *Pseudescherichia*, *Rhodotorula*, *Salinibacterium*, *Sphingobacterium*, *Ustilago*, and *Vogesella*, only one isolate (0.3%) was obtained. Due to the variation in the number of isolates from different soils sampled and the limited number of representative soil types (only two from urban areas and two from lake vicinities compared to five in meadow environments), no correlation could be established between the soil origins and the microbial genera recovered.

The iChip method revealed a higher diversity at the genus level, with isolates from 47 different genera, compared to 38 genera from the DDP method (Figure 3B). Both techniques shared isolates from 20 common genera, representing 83.5% (132 isolates) of the total identified isolates cultivated by DDP and 75% (135 isolates) by the iChip. However, the proportions of common genera differ according to the technique (Figure 2 and Figure 3B). Detailed proportions of the cultivated genera from both methods are shown in Table S3. For example, the iChip seems better adapted for the isolation of *Actinomycetota* (e.g., *Streptomyces, Microbacterium*, and *Micrococcus), Ascomycota*, and *Basidiomycota*, whereas DDP allows the recovery of more *Bacillota* (e.g., *Bacillus, Paenibacillus, Priestia*). Additionally, 45 genera were exclusively recovered by one method but not the other. DDP exclusively yielded 18 genera, representing 16.5% of the isolates recovered by this method, whereas the iChip exclusively yielded 27 genera, representing 25% of the isolates cultivated using the iChip (Figure 3A, B). For isolates identified at the species level, four and seven of them, cultivated by DDP and the iChip, respectively, had a 16S rDNA sequence similarity below the 98.65% threshold for the delineation of new bacterial species, suggesting that they potentially constitute novel species (Table S2) [39]. These isolates belonged to the genera *Flavobacterium* (six isolates), *Massilia* (2 isolates), *Krasilnikoviella, Pedobacter*, and *Sporosarcina* (each with one isolate). Finally, 34 cultivated isolates were affiliated with 22 species recently described (Table S2).

### 3.2. Antibacterial Activity Screening

**Figure 4:**
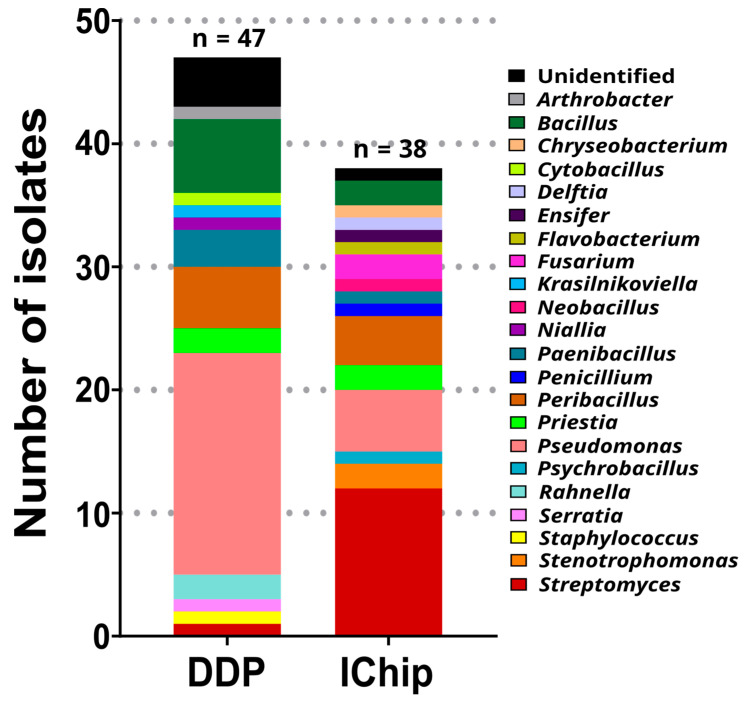
Microbial diversity at the genus level of isolates cultivated by DDP and the iChip and active against at least one target bacteria using a deferred antagonism assay. n: number of active isolates cultivated by each cultivation method.

The cultivated microorganisms were evaluated for antibacterial activity against the indicator microorganisms. Isolates were tested in their recovery medium through a deferred antagonism assay. Of the 386 isolates, 85 (22%) presented antibacterial activity against at least one target microorganism (Table S4). Around 25.8% and 18.6% of the DDP- and iChip-derived isolates showed antibacterial activity. Sixty-one isolates (15.8%) showed antibacterial activity exclusively against the Gram-positive strains and only three (0.8%) exclusively against the Gram-negative strains. Twenty-one isolates (5.4%) showed a broad-spectrum activity, being active against both types of target bacteria. In total, 7 isolates (1.8%) were active against PA, 19 (4.9%) against EC, 11 (2.9%) against KP, 30 (7.8%) against SA, 28 (7.3%) against EF, and 62 (16.1%) against ML. The taxonomic affiliations of the active isolates cultivated by both cultivation methods are shown in Figure 4.

The most active genus was *Pseudomonas* (23 isolates), followed by *Streptomyces* (13 isolates), *Peribacillus* (9 isolates), and *Bacillus* (8 isolates). Moreover, two or more active isolates belonged to the following genera: *Paenibacillus* and *Priestia* (each with four isolates), *Fusarium*, *Rahnella*, and *Stenotrophomonas* (each with two isolates). One isolate was active in each of the genera *Arthrobacter*, *Chryseobacterium*, *Cytobacillus*, *Delftia*, *Ensifer*, *Flavobacterium*, *Krasilnikoviella*, *Neobacillus*, *Niallia*, *Penicillium*, *Psychrobacillus*, *Serratia*, and *Staphylococcus*. Five isolates were unidentified due to low matching occurrence through MALDI-TOF analysis and our inability to obtain PCR products using the described protocol.

**Figure 5:**
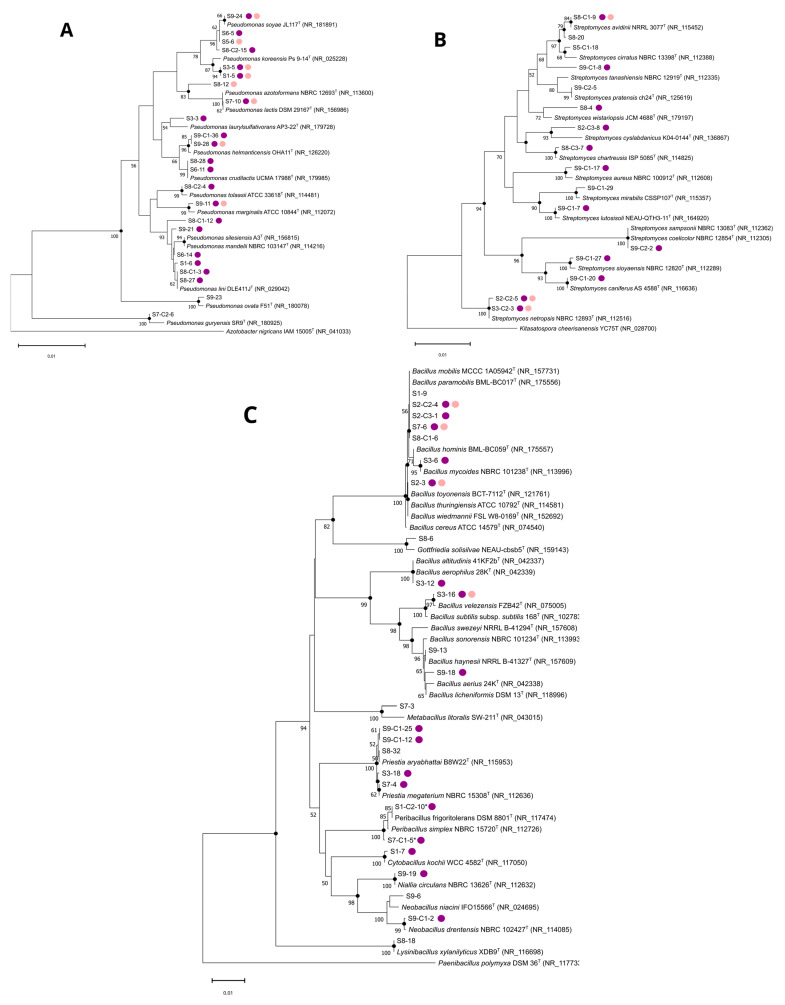
Neighbor-joining phylogenetic trees based on 16S rDNA sequences showing the relationship between sequenced isolates and representatives of the (**A**) *Pseudomonas*, (**B**) *Streptomyce*s, and (**C**) *Bacillaceae* taxa. Because of sequence similarity and clustering in the same node, only 2 of the 14 *Peribacillus* sequenced isolates are shown to simplify *Bacillaceae* tree reading. Bootstrap values (given as percentages of 1000 replicates) >50% are displayed at branch points. Filled black circles indicate that the corresponding nodes were also recovered in the trees generated with the maximum-parsimony algorithm. The type strains *Azotobacter nigricans* IAM 15005^T^, *Kitasatospora cheerisanensis* YC75^T^, and *Paenibacillus polymyxa* DSM 36^T^ were used as outgroups for the *Pseudomonas*, *Streptomyces*, and *Bacillaceae* trees, respectively. Bar, 0.01 substitutions per site. Purple and light pink filled circles indicate antibacterial activity in a deferred antagonism assay against at least one Gram-positive or one Gram-negative target bacteria, respectively.

Phylogenetic trees based on the 16S rDNA of the sequenced isolates and their closest type strains were constructed for the most abundant active representatives, the *Pseudomonas* and *Streptomyces* genera and the *Bacillaceae* family (Figure 5). Phylogenetic constructions showed that some strains (e.g., S7-10, S9-24, S2-C2-5, S1-C2-10, S7-3) have high sequence similarity and branch together with their closest phylogenetic type strain. In contrast, for some strains (e.g., S8-C2-15, S9-28, S9-C1-36, S9-C1-8, S8-20, S6-14, S3-15), new nodes are obtained suggesting divergence between those isolates and their closest phylogenetic type strain emphasizing the need for further analysis to better characterize those isolates and confirm their species affiliation.

To assess the ability of microbial isolates to produce antibacterial compounds, isolates that showed antibacterial activity through the deferred antagonism assay were subjected to liquid fermentation and an agar-well diffusion assay. Of the 85 active isolates, 33 showed continued and secreted antibacterial activity against at least one indicator strain in the fermentation conditions used (Table S5). Among them, ten strains belonged to the genus *Pseudomonas*, six to *Streptomyces*, five to *Bacillus*, and three to *Priestia*. In addition, one strain was active for each genus *Cytobacillus*, *Delftia*, *Flavobacterium*, *Fusarium*, *Paenibacillus*, *Penicillium*, *Peribacillus*, *Stenotrophomonas*, and one unidentified due to a negative PCR product (S9-25).

## 4. Discussion

Culture-independent methods, including metagenomics, have greatly expanded our understanding of the microbial world. A vast, uncharacterized diversity of microorganisms exists in the environment that cannot be readily cultured in the laboratory. However, to better understand those communities, the cultivation of microorganisms remains essential to comprehensively understand their features, such as growth, physiology, metabolism, interactions, and the valuable biomolecules they produce. Assessing these hitherto uncultured microorganisms has received significant interest in recent decades, leading to the elaboration of enrichment strategies [21,23]. Some studies focus on modifying in vitro growth conditions, particularly through changes in culture media composition. Indeed, using diluted or nutrient-poor media has been shown to select previously uncultured bacteria better adapted to oligotrophic conditions, similar to those met in the natural environment [40,41,42,43,44]. Another cultivation approach involves simulating the natural environment. Several in situ cultivation systems have been developed to allow bacterial growth in their natural environmental conditions [25]. Among them, the iChip, derived from diffusion chambers [45], was engineered to cultivate and isolate new microorganisms in a high-throughput manner [26,29] and aimed towards the discovery of new antibiotics [27,46,47].

In our study, we employed the traditional cultivation method (DDP) using rich and diluted media and the iChip approach to enhance the diversity of recovered environmental microorganisms. Our results showed that the combined use of these two cultivation methods on the same soil samples led to a greater diversity of phylogenetically distant microorganisms, spanning 65 genera, compared to using them alone. Specifically, we observed a higher diversity of cultivated microorganisms (total and exclusive genera) with the iChip compared to the DDP method (Figure 3A,B). These observations agree with the known increased capacity of the iChip to access hitherto uncultivated bacteria. Indeed, the use of the iChip was described to enhance microbial recovery by 5- to 300-fold, depending on the study [29,48].

The six microbial phyla recovered in this study are well-known inhabitants of soil environments and have been previously cultivated in other studies [49,50,51,52,53,54]. This result aligns with a study that used both standard culture methods and in situ cultivation with a designed bioreactor system. Indeed, Chaudhary et al. reported the isolation of the same soil bacterial phyla (e.g., *Pseudomonadota, Bacillota, Actinomycetota*, and *Bacteroidota)* in similar proportions [51]. Furthermore, our study showed that more *Actinomycetota* are recovered using the iChip than DDP. This enrichment in *Actinomycetota* isolates can be explained by the limited competitiveness between microorganisms using the iChip compared to DDP. Indeed, *Actinomycetota* representatives generally have longer generation times and can be outcompeted by fast-growing microorganisms in standard plating. In the iChip, the low number of cells in wells allows them to grow without or with limited competition, thus simplifying their isolation. In agreement with a study published by Dos Santos et al., it appears that the iChip can recover many *Actinomycetota* from marine sediments without requiring any particular pre-treatment methods (heat treatment, sample dryness, or antibiotics addition) commonly applied to environmental samples to enrich for these bacteria [55]. This may be of interest in the quest for new antibiotics, as *Actinomycetota* representatives are well-known producers of antibacterial compounds and harbor a sizable repertoire of biosynthetic gene clusters [15,16].

As mentioned, the use of diluted media and the iChip are known to enhance the success of cultivation and the probability of discovering new microorganisms. Based on 16S rDNA similarity, our study allows the recovery of 11 potentially new bacterial species. Most of them were cultivated using the iChip method (seven) and three were obtained using DDP with diluted media and only one by DDP with rich media. Interestingly, six isolates were affiliated with the *Flavobacterium* genus (Table S2). This taxon is known to be a major microbial representative in soil and other environments, and many new species were described in the last decades, increasing from around 30 to more than 300 validly published species between 2006 and 2024 [56,57]. DDP with rich media allowed us to cultivate only one new isolate (S8-9) with the closest known genera/species *Krasilnikoviella muralis* proposed by Nishijima et al. in 2017 [58]. Further investigations are ongoing to determine the novelty of these 11 potentially new species. The novelty in our microbial collection lies mainly at the species level as previously shown [55,59]. Lloyd et al. noticed that most innovative cultivation techniques developed to access uncultured microorganisms allow the recovery of new taxa but more frequently at the species to family levels [20,26,44,51,60]. They suggested that these methods were likely successful at culturing cells from previously cultured clades that are temporally and reversibly recalcitrant to culture, also known as viable but non-culturable cells (VBNCs) [20]. Our study allows the recovery of 34 isolates that are affiliated with relatively new bacterial species (described after 2017), suggesting the growing ability of microbiologists to access non-previously cultured microorganisms.

One objective of this study was to create an environmental microbial collection with untapped or underexplored microorganisms to assess their ability to produce antibacterial compounds. The recovered environmental strains were screened for their ability to inhibit bacterial strains belonging to the priority list of the WHO [4]. Among the 386 microbial isolates, 85 isolates showed activity using the deferred antagonism assay against at least one target strain. As expected, most isolates (61) were specifically active against Gram-positive strains whereas only 3 were found to be specifically active against Gram-negative bacteria. Interestingly, 21 showed broad-spectrum activity. This is consistent with other antibacterial screening studies and explains part of the current difficulty in finding new antibiotics against the growing threat of resistant Gram-negative bacteria.

The observed antibacterial activities are mainly associated with the *Pseudomonas*, *Streptomyces*, and *Bacillaceae* affiliated isolates. These bacterial taxa are well-known antibiotic producers, as are the fungal genera *Penicillium* and *Fusarium*, to which some other active isolates are also affiliated. Among our active isolates from the *Streptomyces* genus, some are closely related to well-known antibiotic-producing species. For example, S9-C2-2 and S3-C2-3 are closely related to *S. coelicolor* and *S. netropsis* (Figure 5B), respectively, known for producing antibiotics such as clorobiocin and actinorhodin, or netropsin and distamycin A [61]. Nevertheless, even in well-known genera or species, new antibacterial compounds are still described [62]. For example, Lacey and Rutledge catalogued 74 novel *Streptomyces* secondary metabolites with a wide variety of chemical scaffolds, in 2020 alone [63]. Other cases obtained within our collection are exemplified by the isolate S3-12 closely related to *Bacillus altitudinis* (Figure 5C). This species has recently been reported to produce pumilarin and altitudin A. However, these two antibiotics did not exhibit activity against *S. aureus* or *E. faecalis*, in contrast to our isolate [64]. Furthermore, some of our active isolates from these taxa are closely related to species not previously described to produce antibacterial compounds. For example, S9-C1-7 is closely related to *Streptomyces lutosisoli* (Figure 5B), a new species described in 2018 and isolated from muddy soil [65]. To our knowledge, this is the first report of antibacterial activity from this species. This is also the case for our active isolates S1-7 and S7-C2-9 that are closely related to *Cytobacillus kochii* (Figure 5C) and *Psychrobacillus* sp. (Table S4), respectively.

By establishing the phylogenetic relationships of the recovered isolates within this study and known antibacterial-producing taxa, it appears that some active isolates are phylogenetically distant from their closest type strains and form a new node in the tree. For example, S9-C1-8, initially affiliated with *Streptomyces tanashiensis* according to BLAST analysis, seems more distant from all other strains (Figure 5B). This is also the case for S8-C2-15 and S8-C1-15 in the *Pseudomonas* tree, which seems phylogenetically isolated (Figure 5A). Further analysis will be required to affiliate those isolates to the species level.

As the phylogenetic analysis used in this study (16S rDNA) is not sufficient to finely define the bacterial species, particularly in these large taxa, further analysis is required to properly identify the isolated strains. For example, Dos Santos et al. showed that their iChip-domesticated *Actinomycetota* strains were genotypically different using PCR fingerprint methods and revealed their potential to produce new antibacterials through a one-strain–many-compounds (OSMAC) approach [54]. Apart from these well-known antibacterial-producing taxa, some active isolates recovered within this study belonged to genera (e.g., *Arthrobacter*, *Chryseobacterium*, *Delftia*, *Ensifer*, *Flavobacterium*, *Rahnella*, and *Stenotrophomonas*) proposed as biocontrol agents in agriculture. Some antibacterial activities have been observed within those genera explaining part of their protective action against phytopathogens [66,67,68,69,70]. However, the bioactive compounds responsible for these observed activities are rarely purified or characterized and are mainly tested against phytopathogens. It is only recently, in the context of searching for new antibiotics, that new compounds have been identified from these underexplored sources. *Delftia* spp. have been shown to produce delftibactin A, a non-ribosomal peptide with broad activity against VRE and MRSA, and against some Gram-negative pathogens like *Acinetobacter baumannii* and KP, but not against EC at tested concentrations [69]. This is different from our isolate S8-C3-9, which show antibacterial activity against EC in the two antibacterial assays but not against SA and EF (Tables S4 and S5), suggesting a potentially different antibacterial compound produced by this strain. Antibacterial activities have also been recently reported from several *Chryseobacterium* species [71,72,73]. This genus, and more largely the *Weeksellaceae* family, has been highlighted as a promising source of new natural products, given the sizable number of biosynthetic clusters encoded in its species’ genomes [74,75]. This suggests the potential of our active isolate S1-C2-6 to produce a new antibacterial compound (Table S4). In addition, due to its novelty, the *Flavobacterium* active isolate S2-C3-2 is also promising for the characterization of a new bioactive compound (Table S4).

By using both DDP with poor and rich medium and in situ cultivation, we create a microbial collection that shows potential for the discovery of new antibacterial agents. The identification of active isolates, the spectrum of activity against our target strains, and comparisons with existing literature now allow us to prioritize certain promising producers for further purification and characterization of the bioactive compounds. Only 33 strains among the 85 active isolates on deferred antagonism assays remained active in the growth condition applied within this study for the well-diffusion assay, complying with the described literature on the requirement to find the condition that activates BCGs. To enhance the production and purification of active compounds, the use of the OSMAC strategy or MATRIX will be implemented in future studies [55,76].

Genome mining has revealed significant uncharacterized biosynthetic potential in several microbial taxa [15,16,17], suggesting that some of our isolates may harbor BGCs that are not expressed or expressed at very low levels under the tested culture conditions, preventing detection of the compounds in our antibacterial assays. To activate those silent BCGs, high-throughput elicitor screening (HiTES) has been proposed by the Seyedsayamdost research group [77]. This approach has been tested on promising microorganisms with a significant number of predicted BGCs per genome (typically > 20), such as *Burkholderia* [77], rare *Actinomycetota* or understudied *Streptomyces* species [78,79], and fungi [80], and showed its ability to activate BCG expression under specific conditions. To better appreciate the potential of our created environmental microbial collection, all non-active isolates have currently entered the bioactivity-HiTES screening protocol [81,82].

Furthermore, the use of tools such as MS-MS dereplication will be implemented to accelerate the identification of bioactive compounds, by enabling the sorting of known compounds from unknown ones [55].

Overall, the present study demonstrates a higher microbial diversity recovery as well as an *Actinomycetota* enrichment using the iChip device. The antibacterial assays performed using this environmental collection allowed us to obtain 18.6% and 25.8% active isolates according to the recovery process. Taken altogether, this microbial collection acts as a seed for further investigation in the quest for new antibacterial compounds.

## Data Availability

The original contributions presented in the study are included in the article/Supplementary Materials, further inquiries can be directed to the corresponding authors.

## Supplementary Materials

The following supporting information can be downloaded at https://www.mdpi.com/article/10.3390/microorganisms12122422/s1, Table S1: Soil samples information; Table S2: Identification of isolated strains; Table S3: Proportions of genera cultivated by the DDP and iChip methods, and the number of active isolates against at least one indicative microorganism in deferred antagonism assays; Table S4: Antibacterial activity of isolates against model bacteria using deferred antagonism assays; Table S5: Antibacterial activity of isolates against model bacteria using agar-well diffusion assays.

## References

[1] Antimicrobial Resistance Collaborators (2022). Global burden of bacterial antimicrobial resistance in 2019: A systematic analysis. Lancet.

[2] GBD 2021 Antimicrobial Resistance Collaborators (2024). Global burden of bacterial antimicrobial resistance 1990–2021: A systematic analysis with forecasts to 2050. Lancet.

[3] O’Neill J. (2016). Tackling Drug-Resistant Infections Globally: Final Report and Recommendations.

[4] World Health Organization (2024). WHO Bacterial Priority Pathogens List, 2024: Bacterial Pathogens of Public Health Importance to Guide Research, Development and Strategies to Prevent and Control Antimicrobial Resistance.

[5] World Health Organization (2022). 2021 Antibacterial Agents in Clinical and Preclinical Development: An Overview and Analysis.

[6] World Health Organization (2024). 2023 Antibacterial Agents in Clinical and Preclinical Development: An Overview and Analysis.

[7] Patridge E., Gareiss P., Kinch M.S., Hoyer D. (2016). An analysis of FDA-approved drugs: Natural products and their derivatives. Drug Discov. Today.

[8] Bérdy J. (2005). Bioactive Microbial Metabolites. J. Antibiot..

[9] Demain A.L. (2014). Importance of Microbial Natural Products and the Need to Revitalize Their Discovery. J. Ind. Microbiol. Biotechnol..

[10] Stennett H.L., Back C.R., Race P.R. (2022). Derivation of a Precise and Consistent Timeline for Antibiotic Development. Antibiotics.

[11] Lewis K. (2020). The Science of Antibiotic Discovery. Cell.

[12] Stokes J.M., Yang K., Swanson K., Jin W., Cubillos-Ruiz A., Donghia N.M., MacNair C.R., French S., Carfrae L.A., Bloom-Ackermann Z. (2020). A Deep Learning Approach to Antibiotic Discovery. Cell.

[13] Ehlert F., Neu H.C. (1987). In Vitro Activity of LY146032 (Daptomycin), a New Peptolide. Eur. J. Clin. Microbiol..

[14] Lewis K. (2012). Recover the lost art of drug discovery. Nature.

[15] Belknap K.C., Park C.J., Barth B.M., Andam C.P. (2020). Genome Mining of Biosynthetic and Chemotherapeutic Gene Clusters in *Streptomyces* Bacteria. Sci. Rep..

[16] Baltz R.H. (2017). Gifted microbes for genome mining and natural product discovery. J. Ind. Microbiol. Biotechnol..

[17] Cimermancic P., Medema M.H., Claesen J., Kurita K., Wieland Brown L.C., Mavrommatis K., Pati A., Godfrey P.A., Koehrsen M., Clardy J. (2014). Insights into Secondary Metabolism from a Global Analysis of Prokaryotic Biosynthetic Gene Clusters. Cell.

[18] Staley J.T., Konopka A. (1985). Measurement of In Situ Activities of Nonphotosynthetic Microorganisms in Aquatic and Terrestrial Habitats. Annu. Rev. Microbiol..

[19] Amann R.I., Ludwig W., Schleifer K.-H. (1995). Phylogenetic Identification and In Situ Detection of Individual Microbial Cells without Cultivation. Microbiol. Rev..

[20] Lloyd K.G., Steen A.D., Ladau J., Yin J., Crosby L. (2018). Phylogenetically Novel Uncultured Microbial Cells Dominate Earth Microbiomes. mSystems.

[21] Lewis W.H., Tahon G., Geesink P., Sousa D.Z., Ettema T.J.G. (2021). Innovations to culturing the uncultured microbial majority. Nat. Rev. Microbiol..

[22] Stewart E.J. (2012). Growing Unculturable Bacteria. J. Bacteriol..

[23] Vartoukian S.R., Palmer R.M., Wade W.G. (2010). Strategies for culture of ‘unculturable’ bacteria. FEMS Microbiol. Lett..

[24] Lagier J.-C., Dubourg G., Million M., Cadoret F., Bilen M., Fenollar F., Levasseur A., Rolain J.-M., Fournier P.-E., Raoult D. (2018). Culturing the human microbiota and culturomics. Nat. Rev. Microbiol..

[25] Schultz J., Modolon F., Rosado A.S., Voolstra C.R., Sweet M., Peixoto R.S. (2022). Methods and Strategies to Uncover Coral-Associated Microbial Dark Matter. mSystems.

[26] Nichols D., Cahoon N., Trakhtenberg E.M., Pham L., Mehta A., Belanger A., Kanigan T., Lewis K., Epstein S.S. (2010). Use of Ichip for High-Throughput In Situ Cultivation of “Uncultivable” Microbial Species. Appl. Environ. Microbiol..

[27] Ling L.L., Schneider T., Peoples A.J., Spoering A.L., Engels I., Conlon B.P., Mueller A., Schäberle T.F., Hughes D.E., Epstein S. (2015). A New Antibiotic Kills Pathogens without Detectable Resistance. Nature.

[28] Shukla R., Peoples A.J., Ludwig K.C., Maity S., Derks M.G.N., de Benedetti S., Krueger A.M., Vermeulen B.J.A., Harbig T., Lavore F. (2023). A New Antibiotic from an Uncultured Bacterium Binds to an Immutable Target. Cell.

[29] Berdy B., Spoering A.L., Ling L.L., Epstein S.S. (2017). In situ cultivation of previously uncultivable microorganisms using the ichip. Nat. Protoc..

[30] Weisburg W.G., Barns S.M., Pelletier D.A., Lane D.J. (1991). 16S ribosomal DNA amplification for phylogenetic study. J. Bacteriol..

[31] White T.J., Bruns T.D., Lee S.B., Taylor J.W., Innis M.A., Gelfand D.H., Sninsky J.J., White T.J. (1990). Amplification and direct sequencing of fungal ribosomal RNA genes for phylogenetics. PCR Protocols: A Guide to Methods and Applications.

[32] Gardes M., Bruns T.D. (1993). ITS primers with enhanced specificity for basidiomycetes—Application to the identification of mycorrhizae and rusts. Mol. Ecol..

[33] Thompson J.D., Higgins D.G., Gibson T.J. (1994). CLUSTAL W: Improving the sensitivity of progressive multiple sequence alignment through sequence weighting, position-specific gap penalties and weight matrix choice. Nucleic Acids Res..

[34] Saitou N., Nei M. (1987). The neighbor-joining method: A new method for reconstructing phylogenetic trees. Mol. Biol. Evol..

[35] Fitch W.M. (1971). Toward Defining the Course of Evolution: Minimum Change for a Specific Tree Topology. Syst. Biol..

[36] Tamura K., Stecher G., Kumar S. (2021). MEGA11: Molecular Evolutionary Genetics Analysis Version 11. Mol. Biol. Evol..

[37] Felsenstein J. (1985). Confidence Limits on Phylogenies: An Approach Using the Bootstrap. Evolution.

[38] Kimura M. (1980). A simple method for estimating evolutionary rates of base substitutions through comparative studies of nucleotide sequences. J. Mol. Evol..

[39] Kim M., Oh H.-S., Park S.-C., Chun J. (2014). Towards a taxonomic coherence between average nucleotide identity and 16S rRNA gene sequence similarity for species demarcation of prokaryotes. Int. J. Syst. Evol. Microbiol..

[40] Connon S.A., Giovannoni S.J. (2002). High-Throughput Methods for Culturing Microorganisms in Very-Low-Nutrient Media Yield Diverse New Marine Isolates. Appl. Environ. Microbiol..

[41] Rappé M.S., Connon S.A., Vergin K.L., Giovannoni S.J. (2002). Cultivation of the ubiquitous SAR11 marine bacterioplankton clade. Nature.

[42] Zengler K., Toledo G., Rappé M., Elkins J., Mathur E.J., Short J.M., Keller M. (2002). Cultivating the uncultured. Proc. Natl. Acad. Sci. USA.

[43] Choi E.J., Nam S.-J., Paul L., Beatty D., Kauffman C.A., Jensen P.R., Fenical W. (2015). Previously Uncultured Marine Bacteria Linked to Novel Alkaloid Production. Chem. Biol..

[44] Janssen P.H., Yates P.S., Grinton B.E., Taylor P.M., Sait M. (2002). Improved Culturability of Soil Bacteria and Isolation in Pure Culture of Novel Members of the Divisions *Acidobacteria*, *Actinobacteria*, *Proteobacteria*, and *Verrucomicrobia*. Appl. Environ. Microbiol..

[45] Kaeberlein T., Lewis K., Epstein S.S. (2002). Isolating “Uncultivable” Microorganisms in Pure Culture in a Simulated Natural Environment. Science.

[46] Quigley J., Peoples A., Sarybaeva A., Hughes D., Ghiglieri M., Achorn C., Desrosiers A., Felix C., Liang L., Malveira S. (2020). Novel Antimicrobials from Uncultured Bacteria Acting against *Mycobacterium tuberculosis*. mBio.

[47] Wirtz D.A., Ludwig K.C., Arts M., Marx C.E., Krannich S., Barac P., Kehraus S., Josten M., Henrichfreise B., Müller A. (2021). Biosynthesis and Mechanism of Action of the Cell Wall Targeting Antibiotic Hypeptin. Angew. Chem. Int. Ed..

[48] Polrot A., Kirby J.R., Olorunniji F.J., Birkett J.W., Sharples G.P. (2022). iChip increases the success of cultivation of TBT-resistant and TBT-degrading bacteria from estuarine sediment. World J. Microbiol. Biotechnol..

[49] Joseph S.J., Hugenholtz P., Sangwan P., Osborne C.A., Janssen P.H. (2003). Laboratory Cultivation of Widespread and Previously Uncultured Soil Bacteria. Appl. Environ. Microbiol..

[50] Furlong M.A., Singleton D.R., Coleman D.C., Whitman W.B. (2002). Molecular and Culture-Based Analyses of Prokaryotic Communities from an Agricultural Soil and the Burrows and Casts of the Earthworm *Lumbricus rubellus*. Appl. Environ. Microbiol..

[51] Chaudhary D.K., Khulan A., Kim J. (2019). Development of a novel cultivation technique for uncultured soil bacteria. Sci. Rep..

[52] Bahram M., Netherway T., Frioux C., Ferretti P., Coelho L.P., Geisen S., Bork P., Hildebrand F. (2021). Metagenomic assessment of the global diversity and distribution of bacteria and fungi. Environ. Microbiol..

[53] Wei H., Peng C., Yang B., Song H., Li Q., Jiang L., Wei G., Wang K., Wang H., Liu S. (2018). Contrasting Soil Bacterial Community, Diversity, and Function in Two Forests in China. Front. Microbiol..

[54] Mhete M., Eze P.N., Rahube T.O., Akinyemi F.O. (2020). Soil properties influence bacterial abundance and diversity under different land-use regimes in semi-arid environments. Sci. Afr..

[55] dos Santos J.D.N., João S.A., Martín J., Vicente F., Reyes F., Lage O.M. (2022). iChip-Inspired Isolation, Bioactivities and Dereplication of *Actinomycetota* from Portuguese Beach Sediments. Microorganisms.

[56] Bernardet J.-F., Bowman J.P., Dworkin M., Falkow S., Rosenberg E., Schleifer K.-H., Stackebrandt E. (2006). The Genus *Flavobacterium*. The Prokaryotes: Volume 7: Proteobacteria: Delta, Epsilon Subclass.

[57] Parte A.C., Sardà Carbasse J., Meier-Kolthoff J.P., Reimer L.C., Göker M. (2020). List of Prokaryotic Names with Standing in Nomenclature (LPSN) Moves to the DSMZ. Int. J. Syst. Evol. Microbiol..

[58] Nishijima M., Tazato N., Handa Y., Umekawa N., Kigawa R., Sano C., Sugiyama J. (2017). *Krasilnikoviella muralis* gen. nov., sp. nov., a Member of the Family *Promicromonosporaceae*, Isolated from the Takamatsuzuka Tumulus Stone Chamber Interior and Reclassification of *Promicromonospora flava* as *Krasilnikoviella flava* comb. nov. Int. J. Syst. Evol. Microbiol..

[59] Zhao J., Shakir Y., Deng Y., Zhang Y. (2023). Use of modified ichip for the cultivation of thermo-tolerant microorganisms from the hot spring. BMC Microbiol..

[60] Bollmann A., Lewis K., Epstein S.S. (2007). Incubation of Environmental Samples in a Diffusion Chamber Increases the Diversity of Recovered Isolates. Appl. Environ. Microbiol..

[61] Moumbock A.F.A., Gao M., Qaseem A., Li J., Kirchner P.A., Ndingkokhar B., Bekono B.D., Simoben C.V., Babiaka S.B., Malange Y.I. (2020). StreptomeDB 3.0: An Updated Compendium of Streptomycetes Natural Products. Nucleic Acids Res..

[62] Culp E.J., Waglechner N., Wang W., Fiebig-Comyn A.A., Hsu Y.-P., Koteva K., Sychantha D., Coombes B.K., Van Nieuwenhze M.S., Brun Y.V. (2020). Evolution-Guided Discovery of Antibiotics That Inhibit Peptidoglycan Remodelling. Nature.

[63] Lacey H.J., Rutledge P.J. (2022). Recently Discovered Secondary Metabolites from *Streptomyces* Species. Molecules.

[64] Lafuente I., Sevillano E., Peña N., Cuartero A., Hernández P.E., Cintas L.M., Muñoz-Atienza E., Borrero J. (2024). Production of Pumilarin and a Novel Circular Bacteriocin, Altitudin A, by *Bacillus altitudinis* ECC22, a Soil-Derived Bacteriocin Producer. Int. J. Mol. Sci..

[65] Shen Y., Sun T., Jiang S., Mu S., Li D., Guo X., Zhang J., Zhao J., Xiang W. (2018). *Streptomyces lutosisoli* sp. nov., a Novel Actinomycete Isolated from Muddy Soil. Antonie Van Leeuwenhoek.

[66] Ramlawi S., Abusharkh S., Carroll A., McMullin D.R., Avis T.J. (2021). Biological and Chemical Characterization of Antimicrobial Activity in *Arthrobacter* spp. Isolated from Disease-Suppressive Compost. J. Basic Microbiol..

[67] Cao Y., Shen Z., Zhang N., Deng X., Thomashow L.S., Lidbury I., Liu H., Li R., Shen Q., Kowalchuk G.A. (2024). Phosphorus availability influences disease-suppressive soil microbiome through plant-microbe interactions. Microbiome.

[68] Chen F., Li J.-Y., Guo Y.-B., Wang J.-H., Wang H.-M. (2009). Biological Control of Grapevine Crown Gall: Purification and Partial Characterisation of an Antibacterial Substance Produced by *Rahnella aquatilis* Strain HX2. Eur. J. Plant Pathol..

[69] Tejman-Yarden N., Robinson A., Davidov Y., Shulman A., Varvak A., Reyes F., Rahav G., Nissan I. (2019). Delftibactin-A, a Non-ribosomal Peptide with Broad Antimicrobial Activity. Front. Microbiol..

[70] Hayward A.C., Fegan N., Fegan M., Stirling G.R. (2010). *Stenotrophomonas* and *Lysobacter*: Ubiquitous Plant-associated Gamma-proteobacteria of Developing Significance in Applied Microbiology. J. Appl. Microbiol..

[71] Dahal R.H., Chaudhary D.K., Kim D.-U., Pandey R.P., Kim J. (2021). *Chryseobacterium antibioticum* sp. nov. with Antimicrobial Activity against Gram-Negative Bacteria, Isolated from Arctic Soil. J. Antibiot..

[72] Tavarideh F., Pourahmad F., Nemati M. (2022). Diversity and Antibacterial Activity of Endophytic Bacteria Associated with Medicinal Plant, *Scrophularia striata*. Vet. Res. Forum.

[73] Chhetri G., Kim I., Kim J., So Y., Seo T. (2022). *Chryseobacterium tagetis* sp. nov., a Plant Growth Promoting Bacterium with an Antimicrobial Activity Isolated from the Roots of Medicinal Plant (*Tagetes patula*). J. Antibiot..

[74] Gavriilidou A., Gutleben J., Versluis D., Forgiarini F., van Passel M.W.J., Ingham C.J., Smidt H., Sipkema D. (2020). Comparative Genomic Analysis of *Flavobacteriaceae*: Insights into Carbohydrate Metabolism, Gliding Motility and Secondary Metabolite Biosynthesis. BMC Genom..

[75] Silva S.G., Homsi M.N., Keller-Costa T., Rocha U., Costa R. (2023). Natural product biosynthetic potential reflects macroevolutionary diversification within a widely distributed bacterial taxon. mSystems.

[76] Liu S.-W., Zhai X.-X., Liu D., Liu Y.-Y., Sui L.-Y., Luo K.-K., Yang Q., Li F.-N., Nikandrova A.A., Imamutdinova A.N. (2023). Bioprospecting of Actinobacterial Diversity and Antibacterial Secondary Metabolites from the Sediments of Four Saline Lakes on the Northern Tibetan Plateau. Microorganisms.

[77] Seyedsayamdost M.R. (2014). High-throughput platform for the discovery of elicitors of silent bacterial gene clusters. Proc. Natl. Acad. Sci. USA.

[78] Xu F., Wu Y., Zhang C., Davis K.M., Moon K., Bushin L.B., Seyedsayamdost M.R. (2019). A genetics-free method for high-throughput discovery of cryptic microbial metabolites. Nat. Chem. Biol..

[79] Zhang C., Seyedsayamdost M.R. (2020). Discovery of a Cryptic Depsipeptide from *Streptomyces ghanaensis* via MALDI-MS-Guided High-Throughput Elicitor Screening. Angew. Chem. Int. Ed..

[80] Lee S.R., Seyedsayamdost M.R. (2022). Induction of Diverse Cryptic Fungal Metabolites by Steroids and Channel Blockers. Angew. Chem. Int. Ed..

[81] Moon K., Xu F., Zhang C., Seyedsayamdost M.R. (2019). Bioactivity-HiTES Unveils Cryptic Antibiotics Encoded in Actinomycete Bacteria. ACS Chem. Biol..

[82] Moon K., Xu F., Seyedsayamdost M.R. (2019). Cebulantin, a Cryptic Lanthipeptide Antibiotic Uncovered Using Bioactivity-Coupled HiTES. Angew. Chem. Int. Ed..

